# Low-dimensional neural ODEs and their application in pharmacokinetics

**DOI:** 10.1007/s10928-023-09886-4

**Published:** 2023-10-14

**Authors:** Dominic Stefan Bräm, Uri Nahum, Johannes Schropp, Marc Pfister, Gilbert Koch

**Affiliations:** 1 Pediatric Pharmacology and Pharmacometrics, University Children’s Hospital Basel (UKBB), University of Basel, Basel, Switzerland; 2 Department of Mathematics and Statistics, University of Konstanz, Constance, Germany

**Keywords:** Pharmacometrics, Pharmacokinetics, Machine learning, Neural ordinary differential equations, Neural networks

## Abstract

Machine Learning (ML) is a fast-evolving field, integrated in many of today’s scientific disciplines. With the recent development of neural ordinary differential equations (NODEs), ML provides a new tool to model dynamical systems in the field of pharmacology and pharmacometrics, such as pharmacokinetics (PK) or pharmacodynamics. The novel and conceptionally different approach of NODEs compared to classical PK modeling creates challenges but also provides opportunities for its application. In this manuscript, we introduce the functionality of NODEs and develop specific low-dimensional NODE structures based on PK principles. We discuss two challenges of NODEs, overfitting and extrapolation to unseen data, and provide practical solutions to these problems. We illustrate concept and application of our proposed low-dimensional NODE approach with several PK modeling examples, including multi-compartmental, target-mediated drug disposition, and delayed absorption behavior. In all investigated scenarios, the NODEs were able to describe the data well and simulate data for new subjects within the observed dosing range. Finally, we briefly demonstrate how NODEs can be combined with mechanistic models. This research work enhances understanding of how NODEs can be applied in PK analyses and illustrates the potential for NODEs in the field of pharmacology and pharmacometrics.

**Supplementary Information:**

The online version contains supplementary material available at 10.1007/s10928-023-09886-4.

## Introduction

Pharmacology and pharmacometrics play an important role in research, development, and application of therapeutics [1]. Pharmacometric analyses, including pharmacokinetic (PK) and pharmacodynamic (PD) analyses, are usually based on describing clinical data through mathematical-statistical models with well-defined differential equations based on law-of-mass action and first principles [2–4].

Recent research papers propose machine learning (ML) as a tool complementing conventional pharmacometric methods [5–10]. Several ML approaches were applied to develop predictive models, e.g., to forecast risk of phototherapy in newborns with hyperbilirubinemia [10], to differentiate between diabetes insipidus and primary polydipsia [11], and to facilitate covariate screening and selection [12, 13]. A main method in ML is a neural network (NN), which is basically an approximation approach for non-linear functions. Due to their capability to approximate various input–output relationships, NNs were applied in different scenarios such as (i) data imputation of missing covariates [14], (ii) covariate selection [15], and (iii) model reduction of quantitative systems pharmacology models [8]. Other publications discuss the use of NNs to approximate PK functions for concentration–time profiles and the possibility to perform PK simulations [16].

The substantially different character of most ML approaches compared to conventional PK approaches impede their broad application in pharmacometrics. Therefore, the recently presented approach of neural ordinary differential equations (NODE) [17] gained special attention. In this approach, ordinary differential equations (ODE) are combined with NNs. Although their functionality is similar to ODEs, the right-hand side of an NODE is no longer a mechanism developed by the modeler but an NN that learns the mechanism solely based on available data. It has been shown that NODEs are able to fit PK and PD data as well as other dynamic systems from health sciences well [18–22]. However, despite the shared concepts between ODEs and NODEs, several aspects of NODEs differ from classical modeling with ODEs. Compared to previous publications utilizing NODEs in PK, we aim at developing a pharmacometric based ML model and present a low-dimensional NODE approach related to PK principles.

This research work includes three major parts. First, we build a theoretical concept for our NODEs. To this end, we developed an NODE structure explicitly based on PK principles. This approach differs significantly from most conventional ML approaches, where the structure is developed empirically [23]. With our development, we ensure that the NNs in the NODEs can describe various PK scenarios. Additionally, we discuss opportunities to combine partially known mechanisms with NNs, a discipline called scientific ML [24, 25]. Second, we develop a methodological setup for applying our NODE structures in pharmacometrics. Here, we face common ML challenges, such as avoiding overfitting and performing simulations for unseen data, and provide practical solutions to these challenges. Third, we utilize previously elaborated concepts and setups for the application of our NODEs in pharmacometrics and present the results for various PK scenarios.

The overarching goal of this research work is to provide a general insight into NODEs to further stimulate research and applications in the field of pharmacology and pharmacometrics. The presented low-dimensional NODE concept differs significantly from other NODE implementations including e.g., encoder-decoder structures [19, 26]. It does not include inter-individual variability and covariate effects, which will be subject of further investigation.

### Theoretical

The aim was to develop an NODE structure that is tailored to handle various linear and non-linear PK behavior including distribution processes and potentially delayed absorption. As this paper aims at introducing a general concept, we currently only focus on fitting the average profile of a population and no covariate effects were included. In this section, we focus on theoretical concepts of NODEs in pharmacometrics, particularly PK analyses, which will be presented in five sections. First, a brief introduction to NNs and their characteristic as function approximators is provided. Second, the concept of substituting the right-hand side of an ODE with an NN is presented. Third, the reduction of multi-dimensional ODE systems to a one-dimensional system is presented. This means that there are no assumptions about the mechanism required (e.g., number of peripheral compartments). Fourth, specific NODE structures based on PK principles are developed such that they can be applied to various PK scenarios. Fifth, the concept of combining partially known mechanisms with NNs is briefly addressed. Throughout all presented concepts, single-dose scenarios were considered. Possible adjustments for multi-dose scenarios are provided in the discussion. In addition to the presented NODE related concepts, general ML concepts, such as hyperparameter tuning and cross-validation should be applied if required in specific real-life projects [27, 28].

### Introduction to neural networks

An NN is a parameter-dependent function that characterizes an input–output relationship. The input–output relationship is based on compositions of serial calculation steps, referred as layers. Each layer consists of several neurons and each neuron is a simple calculation step of multiplication and addition [8]. We focus on NNs consisting of one hidden layer $H\in {\mathbb{R}}^{{n}_{Hid}}$ with ${n}_{Hid}$ neurons, and an input feature $X\in {\mathbb{R}}^{{n}_{In}}$ that is mapped to an output feature $Y\in {\mathbb{R}}^{{n}_{Out}}$ with the NN ${f}_{NN} : {\mathbb{R}}^{{n}_{In}}\to {\mathbb{R}}^{{n}_{Out}}$. Hence, the NN structure reads

1 $${f}_{NN}\left(X\right)={\sigma }^{(2)}\left({W}^{(2)}\cdot {\sigma }^{(1)}\left({W}^{(1)}\cdot X+{b}^{(1)}\right)+{b}^{(2)}\right)$$

where ${W}^{(1)}\in {\mathbb{R}}^{{n}_{Hid},{n}_{In}}$ are the weights from the input layer to the hidden layer, ${b}^{(1)}\in {\mathbb{R}}^{{n}_{Hid}}$ the biases at the hidden layer, ${W}^{(2)}\in {\mathbb{R}}^{{n}_{Out},{n}_{Hid}}$ the weights from hidden layer to output layer, ${b}^{(2)}\in {\mathbb{R}}^{{n}_{Out}}$ the biases at the output layer, ${\sigma }^{(1)} : {\mathbb{R}}^{{n}_{Hid}}\to {\mathbb{R}}^{{n}_{Hid}}$ the activation function from input to hidden layer, and ${\sigma }^{(2)} : {\mathbb{R}}^{{n}_{Out}}\to {\mathbb{R}}^{{n}_{Out}}$ the activation function from hidden to output layer. Both activation functions are applied component-wise at the right-hand side of Eq. (1). During the training of an NN, the parameters are optimized in order to approximate the underlying function characterizing the input–output relationship observed in the training data. The advantage of an NN is that, based on some assumptions, even an NN with one hidden layer is capable of approximating any continuous input–output [29].

For illustration purpose, we present an NN structure with ${n}_{In}=1$ and ${n}_{Out}=1$, the prominent non-linear ReLU activation function from input to hidden layer

$${\sigma }^{\left(1\right)}\left(z\right)=\mathrm{max}\left(0, z\right) ,$$

and the identity from hidden to output layer

$${\sigma }^{(2)}\left(z\right)=z$$

The NN in Eq. (1) can be reformulated and the matrix multiplications can be written as the following summation:

2 $${f}_{NN}\left(x\right)=\sum_{i=1}^{{n}_{Hid}}{w}_{1,i}^{(2)}\cdot \mathrm{max}\left(0,{w}_{i,1}^{(1)}\cdot x+{b}_{i}^{(1)}\right)+{b}^{(2)} ,$$

where the indices denote the entries of the matrices (e.g., ${w}_{i,1}^{(1)}$ the i’th entry of the weight matrix ${W}^{(1)})$. We observe that an NN is basically a summation of activation functions and that in this case, the output is a stepwise linear function.

**Fig. 1:**
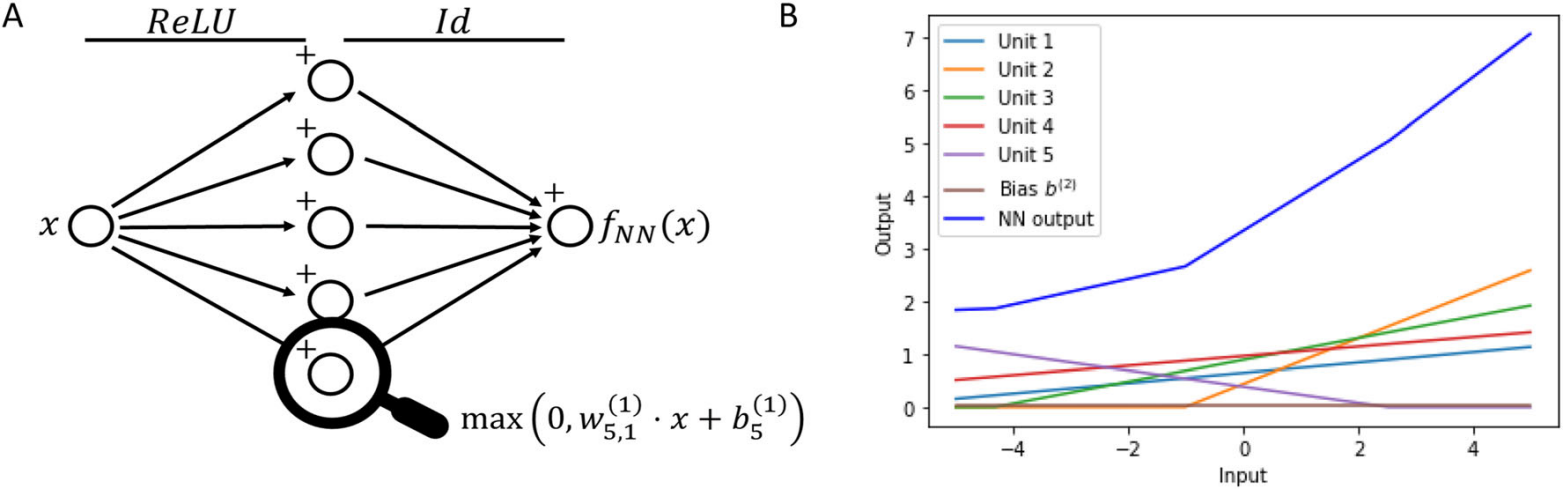
In panel **A**, the structure of an NN with a one-dimensional input, one hidden layer with five neurons, and a one-dimensional output is shown. Arrows denote multiplication with weights, plus signs denote addition of biases and ReLU and Id indicate the applied activation function from input to hidden layer and from hidden to output layer, respectively. In panel **B**, outputs of the neurons in the hidden layer and the final output of the NN in panel A is illustrated

In Fig. 1A, we present a schematic NN with ${n}_{Hid}$ = 5. In Fig. 1B, we show an example of the unit activations in the hidden layer and its resulting NN output.

### Introduction to neural ODEs

The basic concept of NODEs is based on ODEs expressing the derivative of a variable $x$ as an explicit function $f\left(x\right)$ as follows:

3 $$\frac{d}{dt}x=f\left(x\right) , \,x\left(0\right)={x}^{0} .$$

In the previous section, NNs were presented as function approximators. In NODEs, NNs are utilized to approximate the right-hand side of an ODE. Hence, the function $f\left(x\right)$ from Eq. (3) is now substituted with an NN, namely ${f}_{NN}\left(x\right)$. This results in

4 $$\frac{d}{dt}x={f}_{NN}\left(x\right) , \,x\left(0\right)={x}^{0} .$$

The NODE in Eq. (4) can then be solved with any ODE-solver, like the ODE in Eq. (3), and the NN parameters of ${f}_{NN}\left(x\right)$ are optimized based on training data. Thus, NODEs are a data-driven approach to approximate the dynamics observed in training data, such as PK concentration–time data.

### Reduction of multi-dimensional systems to a non-autonomous one-dimensional system

PK models are usually autonomous multi-dimensional ODE systems, meaning the right-hand side of the ODE is time-independent. The reason for this multi-dimensionality is the characterization of the underlying pharmacological, physiological, and biological mechanism based on first principles and law-of-mass action. For example, a two-compartment intravenous (IV) model consists of two equations, one for the central compartment fitted against the measured concentration data, and one for the peripheral compartment. Another example are oral (PO) models with delayed absorption, i.e., including one or multiple transit compartments. An even more complex example is the target-mediated drug disposition (TMDD) IV model [30] with three equations (central compartment, receptor, and drug-receptor complex).

**Fig. 2:**
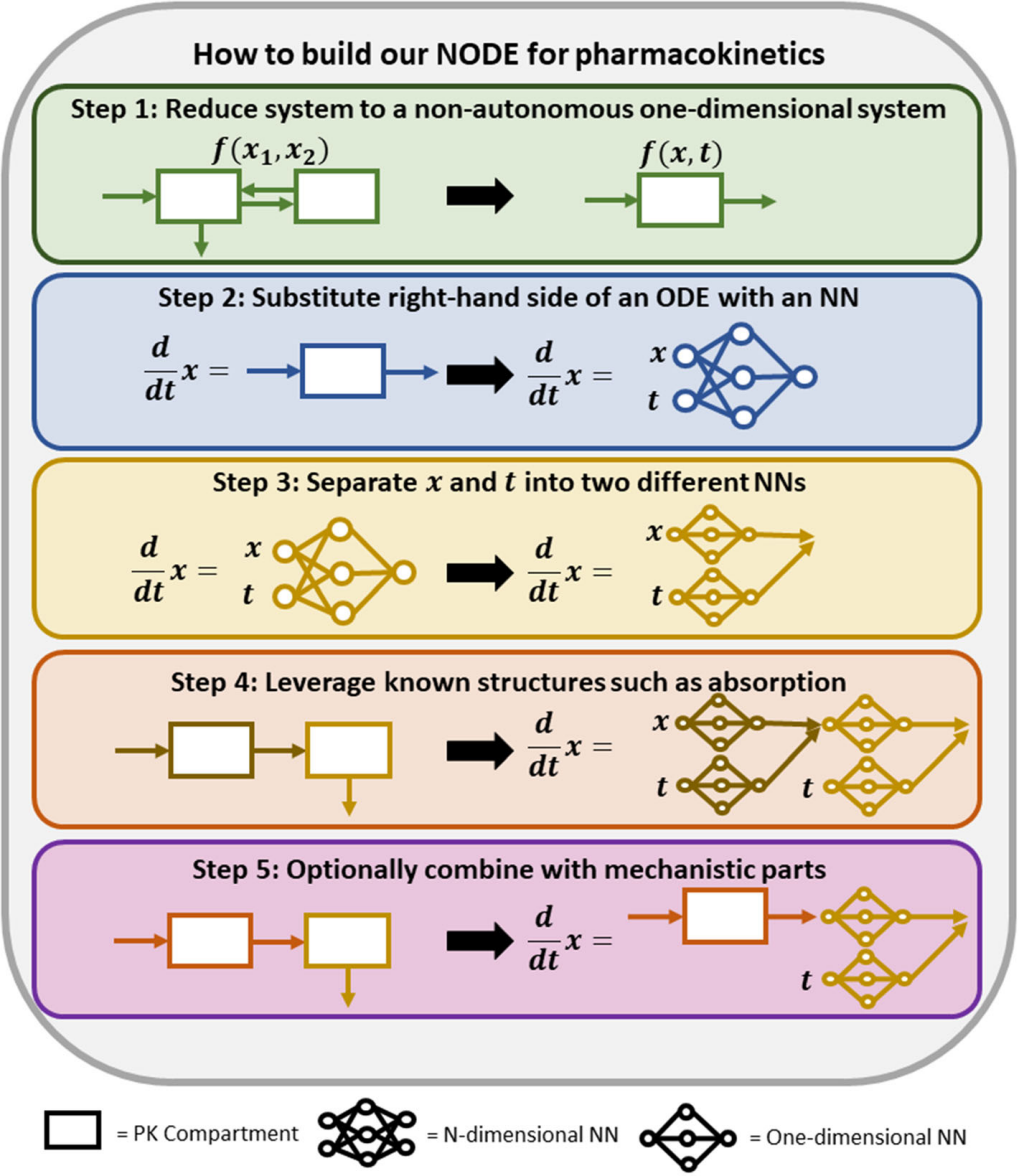
A schematic overview of the major concepts to build our NODE for PK. The first step towards our NODE for PK is to reduce autonomous (time-independent right-hand side) multi-dimensional ODE systems to a non-autonomous (time-dependent right-hand side) one-dimensional system since the general NODE should be applicable without mechanistic assumptions. The second step is to substitute the right-hand side of an ODE with an NN. In a third step, the concentration variable and time variable were separated into two NNs with one-dimensional input and output. In a fourth step, generally known structures such as drug administration with absorption process can be included in the NODE. In a fifth step, the NODE can optionally be combined with a known mechanistic part, if prior knowledge is available

In contrast, the advantage of NODEs is that they are a data-driven approach. Thus, no assumptions about the mechanistic model, e.g., the number of transit or peripheral compartments, should be required. To this end, we reduced multi-dimensional ODE systems to a one-dimensional ODE system. As shown for example for linear ODE systems in Appendix A1 and A2, this dimensional reduction of autonomous multi-dimensional systems results in non-autonomous systems, i.e., the function on the right-hand side is time-dependent, as indicated in step 1 of Fig. 2.

#### Basic NODE structure of a non-autonomous one-dimensional system

As indicated in step 2 of Fig. 2 and based on the reduction of autonomous multi-dimensional systems to a non-autonomous one-dimensional system, our basic NODE structure, e.g., capable to fit IV PK data without assumptions about the number of peripheral compartments, reads

5 $$\frac{d}{dt}{x}_{C}={f}_{NN}^{C}\left({x}_{C},t\right) , \,{x}_{C}\left(0\right)=\frac{d}{V} ,$$

where we emphasize with ${x}_{C}$ the “central compartment”, i.e., the state variable that is fitted against the concentration measurements. In addition, $t$ denotes the explicit time, $d$ the dose and $V$ the volume of distribution. The volume of distribution $V$ can be considered as a special PK parameter since it can appear in the initial condition of the PK model, compare Eq. (5), and basically scales the PK profile. Therefore, the estimated parameters are the NN parameters and $V$, if not stated otherwise.

### Development of our NODE based on PK principles

In this section, several PK principles are utilized to adjust and simplify our NODE structure for the application of NODEs tailored to PK scenarios.

#### General NODE structure with separated time- and concentration-dependent right-hand side

As presented in Eq. (5), a one-dimensional NODE to fit IV PK data must be non-autonomous. In contrast to the formulation in Eq. (5), however, the concentration-dependent and time-dependent functions are additively separated, according to Appendix A1 and A2. Therefore, and with additional motivation in Appendix A4, the inputs concentration and time were separated into two NNs with one-dimensional input and output in our NODE structure. As indicated in step 3 of Fig. 2, this results in our general NODE structure with a concentration-dependent NN, ${f}_{NN}^{C1}\left({x}_{C}\right)$, and a time-dependent NN, ${f}_{NN}^{C2}\left(t\right)$, according to

6 $$\frac{d}{dt}{x}_{C}={f}_{NN}^{C1}\left({x}_{C}\right)+\frac{d}{V}\cdot {f}_{NN}^{C2}\left(t\right) , \,{x}_{C}\left(0\right)=\frac{d}{V} .$$

Like observed, e.g., after the completion of distribution processes, and to assure that for $t\to \infty$ the state variable ${x}_{C}$ does not increase infinitely, the time-dependency in the NODE should vanish. To this end, we restrict the weights from input to hidden layer in ${f}_{NN}^{C2}$ to be as ${w}_{i,1}^{(1)}$ < 0, such that $\underset{t\to \infty }{\mathrm{lim}}\mathrm{max}\left(0,{w}_{i,1}^{(1)}\cdot t+{b}_{i}^{(1)}\right)=0$ and therefore $\underset{t\to \infty }{\mathrm{lim}}{f}_{NN}^{C2}\left(t\right)={b}^{(2)}$. This was achieved by applying ${w}{\prime}=-({w}^{2})$ where $w$ indicates the original weight and $w{\prime}$ the restricted weight applied in the NN. The term $\frac{d}{V}$ in Eq. (6) is required to fit different dose levels with the same NODE.

#### NODE structure with absorption

In principle, Eq. (6) can produce non-monotonic behavior, as observed e.g., for PO administered drugs. Since the route of administration is known from the clinical setup, we apply the typical absorption structure and add an additional absorption compartment ${x}_{A}$ to Eq. (6), as illustrated in step 4 of Fig. 2. Hence, we obtain the two-dimensional NODE

7 $$\frac{d}{dt}{x}_{A}=d\cdot {f}_{NN}^{A1}\left(t\right)-{f}_{NN}^{A2}\left({x}_{A}\right) , \,{x}_{A}\left(0\right)=0 ,$$

8 $$\frac{d}{dt}{x}_{C}={f}_{NN}^{A2}\left({x}_{A}\right)-{f}_{NN}^{C1}\left({x}_{C}\right) -d\cdot {f}_{NN}^{C2}(t) , \,{x}_{C}\left(0\right)=0 ,$$

where we set the weights from input to hidden layer as ${w}_{i,1}^{(1)}$ < 0 in ${f}_{NN}^{A1}$ and ${f}_{NN}^{C2}$. In case of an absorption compartment, we omit $V$ since scaling can take already place in ${f}_{NN}^{A2}$. Motivated by Appendix A2, the time-dependent NN in the absorption compartment ${f}_{NN}^{A1}\left(t\right)$ allows to also model PO administered drugs with delayed absorption.

#### NODE structure with infusion

Like the NODE structure with absorption, IV infusion can be explicitly built into the NODE since the route of administration, the infusion rate ${k}_{in}$, and infusion time ${t}_{inf}$ are known from the clinical setup. Thus, Eq. (6) can be modified like a conventional ODE for IV infusion

9 $$\frac{d}{dt}{x}_{C}=\frac{{k}_{in}}{V}\cdot 1\left(t\le {t}_{inf }\right)+{f}_{NN}^{C1}\left({x}_{C}\right) +\frac{d}{V}\cdot {f}_{NN}^{C2}\left(t\right), \,\,{x}_{C}\left(0\right)=0 .$$

Note that $V$ must be estimated since the input to the central compartment is explicitly built in with ${k}_{in}$ and in contrast to the absorption in Eq. (8), the scaling cannot be approximated by an NN. Dose $d$ is calculated as the amount of total drug applied, i.e., $d={k}_{in}\cdot {t}_{inf}$.

### Combining partially known mechanisms with neural networks

NODEs can be combined with partially known mechanisms, as illustrated in step 5 of Fig. 2. Combining known mechanistic parts with NNs that are supposed to learn the unknown parts, called scientific machine learning [24, 25], is currently strongly evolving. Here, we only scratch on the surface of this discipline and present for illustration purpose two potential PK examples.

#### NODE structure with mechanistic elimination

Even though an NODE with Eqs. (7, 8) can fit various oral PK models, prior knowledge of the drug of interest can be leveraged. Assuming a one-compartment model, e.g., from a previous IV study, with a known elimination mechanism and an unknown absorption mechanism, Eqs. (7, 8) can be modified to

10 $$\frac{d}{dt}{x}_{A}=d\cdot {f}_{NN}^{A1}\left(t\right)-{f}_{NN}^{A2}\left({x}_{A}\right) , \,{x}_{A}\left(0\right)=0 ,$$

11 $$\frac{d}{dt}{x}_{C}={f}_{NN}^{A2}\left({x}_{A}\right)-{k}_{el}\cdot {x}_{C} , \,{x}_{C}\left(0\right)=0 .$$

Note that this would also be applicable, if linear elimination with unknown distribution mechanism is assumed, replacing the concentration-dependent NN ${f}_{NN}^{C1}\left({x}_{C}\right)$ in Eq. (6) by $-{k}_{el}\cdot {x}_{C}$.

#### NODE structure with mechanistic absorption

Like Eqs. (10, 11), a known linear absorption mechanism but with unknown distribution and elimination mechanism can be assumed by modifying Eqs. (7, 8) to

12 $$\frac{d}{dt}{x}_{A}=-{k}_{a}\cdot {x}_{A} , \,{x}_{A}\left(0\right)=\frac{d}{V} ,$$

13 $$\frac{d}{dt}{x}_{C}={k}_{a}\cdot {x}_{A}-{f}_{NN}^{C1}\left({x}_{C}\right)-\frac{d}{V}\cdot {f}_{NN}^{C2}\left(t\right) , \,{x}_{C}\left(0\right)=0 .$$

## Methods

In this section, essential concepts and setups for NODE applications in pharmacometrics are discussed and solved. The aim is to develop a methodological setup for the application of previously presented low-dimensional NODE structures in various PK analyses. To this end, we address four concepts for NODEs. First, we present how the derivative versus state plot can be utilized to assess properties of trained NODEs. Second, the phenomenon of overfitting is discussed and a solution to avoid overfitting when applying NODEs to PK data is suggested. Third, the challenge of performing simulations with NODEs for unseen data, so-called extrapolation, is investigated and corresponding scenarios are discussed. Fourth, two loss functions utilized in further applications are presented.

### Utilizing the derivative versus state plot to assess simulation properties of a trained NODE

NNs are optimized only on the data they were trained on and caution is necessary when utilizing NODEs for extrapolation, i.e., simulations beyond the properties available in the trained data. To understand and assess the simulation capability of applied NODEs, we utilize the derivative versus state plot. In this plot, the x-axis corresponds to concentration ${x}_{C}$ and the y-axis is the right-hand side/derivative $\frac{d}{dt}{x}_{C}$. Hence, this plot allows to assess the learned mechanism in a trained NODE.

**Fig. 3:**
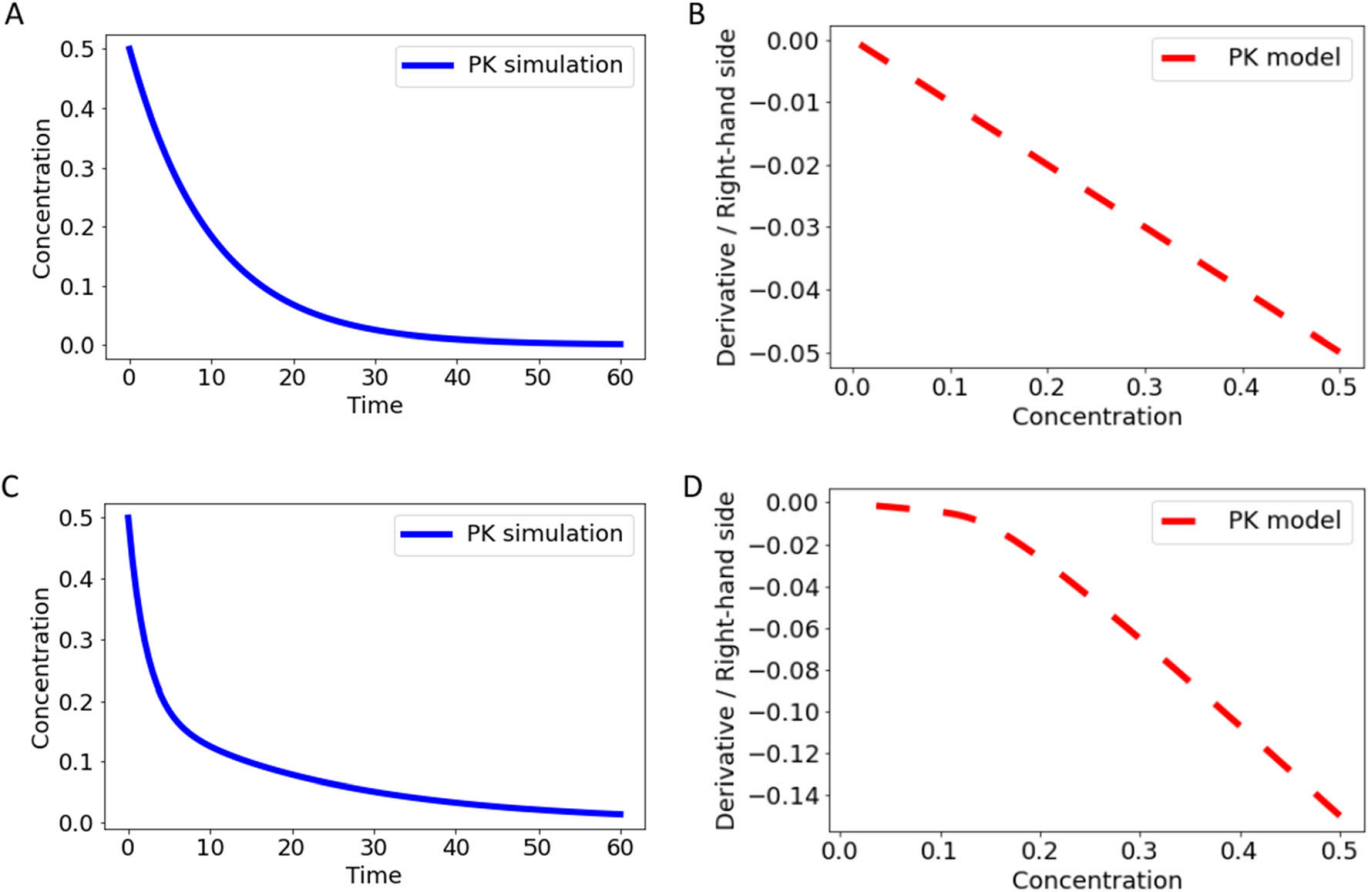
In panel **A** and **C**, simulated concentration–time data is shown for a linear one- and a two-compartment ODE model, respectively. In panel **B** and **D**, the corresponding derivative versus state plots are presented, illustrating the derivative against the concentration

To motivate the derivative versus state plot, we consider two ODE-based examples, a linear one-compartment IV model, and a linear two-compartment IV model. In Fig. 3A, the concentration versus time profile is shown for the one-compartment ODE model. In Fig. 3B, the derivative versus state plot is shown, and we observe the expected linear relationship between ${x}_{C}$ and $\frac{d}{dt}{x}_{C}$. In Fig. 3C, the concentration versus time profile of the two-compartment ODE model is visualized, and in Fig. 3D we observe the expected stepwise linear derivative versus state behavior with a steep slope in the distribution phase transitioning into a flatter slope of the terminal elimination phase.

Hence, plotting the derivative versus state of a trained NODE allows to assess how well the mechanism was learned. In the following, the derivative versus state plot is applied to identify overfitting and to reveal simulation performance for concentrations outside the training range.

### Dealing with overfitting in NODEs

In this section, an example of overfitting and a solution to overcome this problem is presented. The term overfitting is used when a statistical/mathematical model fits the training data well but does not generalize well to new data. Overfitting is more likely to happen when a method has a high degree of freedom, i.e., high number of model parameters compared to available data points.

The highly flexible NODE might not only characterize the underlying mechanism in the data but it might assume that the observed noise (i.e., residual errors) in the training data is part of the mechanism as well. As an example, single concentration measurements at 5 time points were generated with a one-compartment ODE model where the elimination rate was ${k}_{el}$ = 0.1, the volume of distribution $V$ = 2 and the dose $d$ = 1. This data was refitted twice, first with the original ODE model, and second, with the general NODE Eq. (6). The number of parameters in the NODE is much larger with respect to the classical approach and hence, the chance for overfitting is higher.

**Fig. 4:**
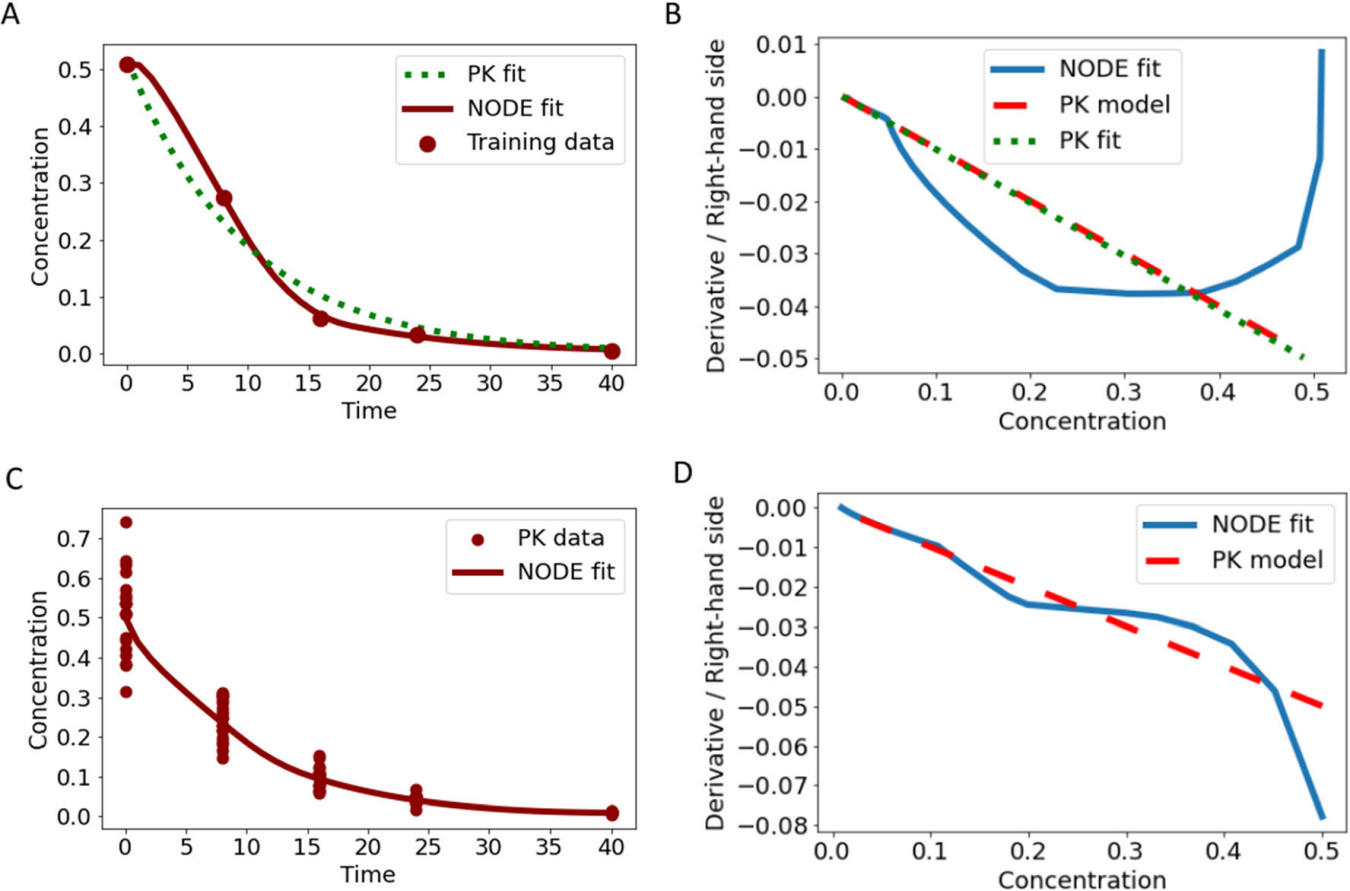
In panel **A**, the fit of the NODE Eq. (6) (red solid line) and the original one-compartment ODE model (green dotted line) to training data (red circles) from single measurements is shown. In panel **B**, the corresponding derivative versus state plot is presented, highlighting the flexibility and indicating overfitting for the NODE. In panel **C** and **D**, pooled training was performed for the NODE Eq. (6), indicating reduced overfitting

Utilizing the NODE, we had a smaller mean squared error of $MS{E}_{NODE}$= 1e−5 compared to the original ODE model with $MS{E}_{PK}$ = 8e−4, as can be observed in Fig. 4A. This is a classical overfitting phenomenon. Basically, the NODE fits not only the data but also the residual errors. This becomes even more clear in Fig. 4B, where the difference of the derivative of the NODE compared to the derivative of the original ODE model is visualized. In a one-compartment ODE model, an approximately linear derivative with constant slope is expected. However, we observe that the NODE produced a derivative deviating completely from the expected derivative and shows unrealistic behavior for a PK scenario. This explains the “better” fit in terms of $MSE$ and highlights the flexibility of NODEs. However, this results in the undesired overfitting.

There are two main solutions to avoid overfitting: reducing the number of parameters in the model or using more data such as multiple measurements per time point. Following the first approach, a strong reduction of the flexibility of the NODE can be obtained by strongly reducing the number of hidden neurons. Hence, we obtained an $MSE$ which is rather comparable to the $MSE$ of the fit with the original ODE model, indicating that the overfit on the data was reduced. However, also the capability of the NODE to handle more intricate PK profiles was strongly impaired. The second approach, which is the one that we are going to focus on in the following, was to use more data with similar sampling scheme and perform pooled training for the NODE. Due to the similar sampling scheme and the pooled training, the residual errors from multiple data points cancel each other out so that the NODE only fits the underlying mechanism. This can be nicely seen in Fig. 4C, where the data and fit of the NODE Eq. (6) is shown for ${n}_{Hid}$ = 20 and no overfitting was observed, i.e., the NODE behaves like the original ODE model, compare Fig. 4D. Note that deviations in the derivative versus state plot may appear large but only small deviations are observed in the predictions. Hence, the learned mechanism is closer to the underlying mechanism and not falsified by overfitting. In all following examples, the solution to utilize pooled training is applied to avoid overfitting. Note that with the pooled training approach, there are still only the average dynamics modeled and no information about inter-individual variability is gained. Whether it is feasible to collect more data might be project-dependent and reducing the number of parameters might be preferred in some analyses.

### Performing simulations with NODEs for unseen data (extrapolation)

In this section, the limited ability of NNs to generalize to data outside of the training range and consequently the impact on NODEs is demonstrated. This means for PK applications that simulations of concentrations outside the trained range can lead to incorrect results.

**Fig. 5:**
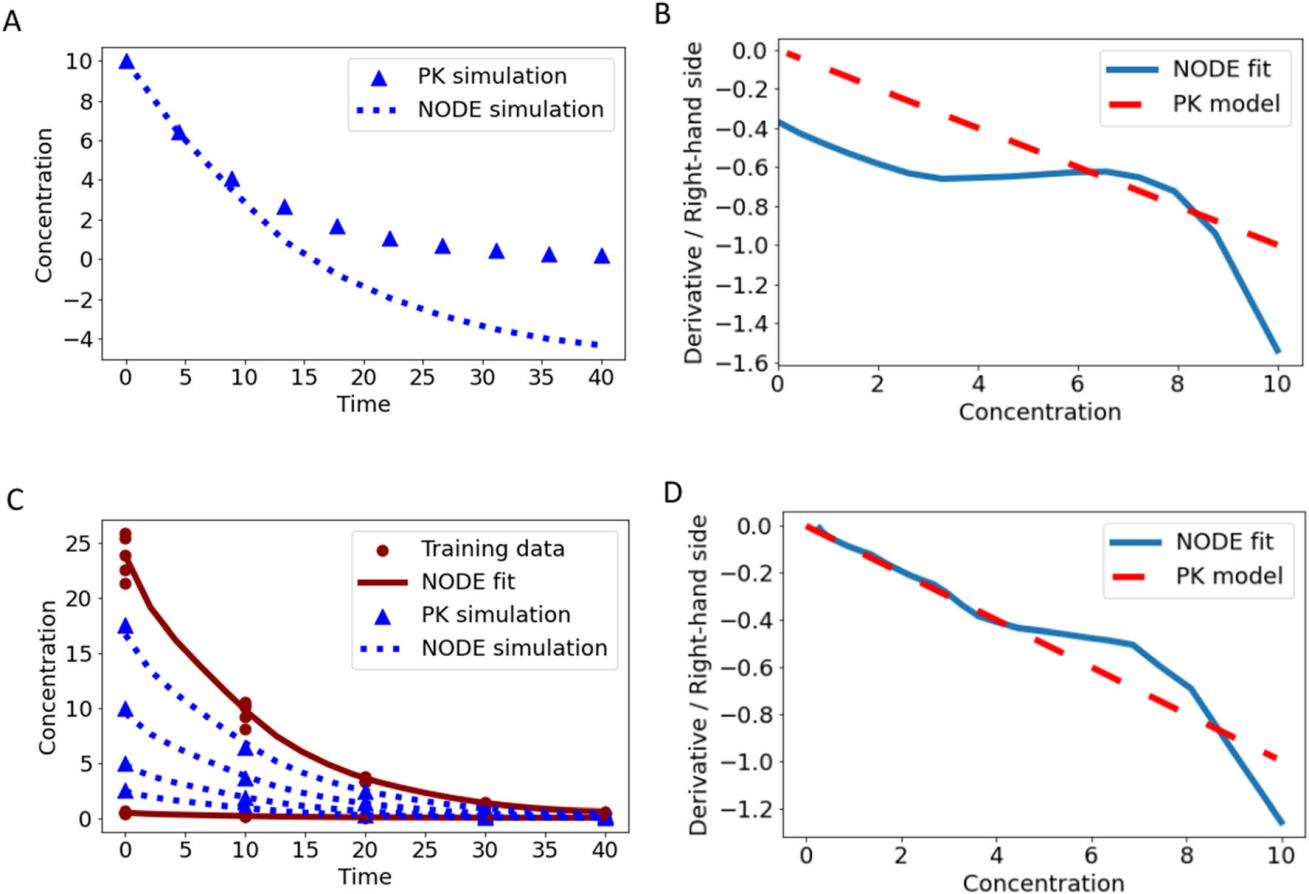
In panel **A**, simulated data (blue triangles) for dose $d$ = 20 with the original ODE model and the NODE Eq. (6) trained on a dose $d$ = 1 (dashed blue line) is shown. In panel **B**, the corresponding derivative versus state plot indicates the inability of NNs to extrapolate which inherits to the NODE. In panel **C** and **D**, simulated data for different unseen doses ($d$ = 5, 10, 20 and 35) with the original ODE model and the NODE Eq. (6) trained on two dose levels $d$ = 1 and $d$ = 50 is shown and the corresponding plots indicate the interpolation capability of NNs

To illustrate this phenomenon, we apply the trained NODE Eq. (6) based on the data for one dose level, $d$ = 1, from the previous paragraph. But now, we compare simulations of the NODE for a new dose, $d$ = 20, with the original ODE model. The discrepancy between the ODE and NODE simulations are shown in Fig. 5A, where unrealistic concentrations even with negative values were generated. In Fig. 5B, the difference of the derivative between the NODE fit and the original ODE model is shown. As expected, the NODE learned the mechanism only for the dose it was trained on.

As a solution we suggest to train the NODE with data produced with a low dose ($d$ = 1) and a high dose ($d$ = 50) and to exploit the interpolation capability of NNs. As presented in Fig. 5C, simulations with the NODE for doses in this range, i.e., $d$ = 5, 10, 20 and 35 very well coincide with the original ODE model even though theses doses were not explicitly introduced to the NODE (compare Fig. 5D). This suggests that the mechanism in this dose range was learned. This approach of fitting two doses to cover the range between them will be followed in the next examples.

### Applied loss functions for data fitting

The loss function (in classical approaches usually referred as objective function) describes the difference between the prediction and the observed data. Using the loss function, the gradients for the model parameters (weights and biases) are computed in the process of backpropagation. As NODEs are a data driven approach, the choice of the loss function strongly influences what part of the data is the main driver of the optimization. If the observed values are in a similar range and no outliers are observed, one of the most common loss functions is the Mean Squared Error (MSE)

14 $$MSE=\frac{1}{m\cdot n}\sum_{i=1}^{m}\sum_{j=1}^{n}{\left({x}_{C}\left({t}_{i}\right)-{x}_{ji}\right)}^{2} ,$$

where $m$ denotes the number of measurement time points, $n$ the number of measurements per time point, ${x}_{C}({t}_{i})$ the predicted value at time point ${t}_{i}$ and ${x}_{ji}$ the j`th observed value at time point ${t}_{i}$.

In the case of larger ranges, the NODE with an MSE as loss function would poorly learn smaller observations because the residual errors of the high values would dominate the MSE. In such cases, the Weighted Mean Squared Error (WMSE) was applied according to

15 $$WMSE=\frac{1}{m\cdot n}\sum_{i=1}^{m}\sum_{j=1}^{n}\frac{{\left({x}_{C}\left({t}_{i}\right)-{x}_{ji}\right)}^{2}}{{x}_{ji}} .$$

In the following applications of NODEs, the MSE was applied for data fitting, if not indicated otherwise.

Note that another approach for dealing with large ranges would be to fit data in log-scale. This would bring the additional benefit of restricting the predictions to the positive value range. However, for simplicity, especially when combining the NN with mechanistic parts as described in step 5 of Fig. 2, we decided to stay in the normal scale in the following applications.

### Remarks about numerical implementation of NODEs

All calculations were done within Python. The training data was generated by numerically integrate the ODE using the package Scipy [31] and by adding a proportional residual error. For the NN framework, the package PyTorch [32] was applied and for the optimization of these NNs within an ODE-solver the package Torchdiffeq [17] was utilized. Note that in our implementation, the classical approach of sequential matrix multiplications was used, as shown in Eq. (1), and not the summation form in Eq. (2). In all examples, the Adam optimizer [33] was used with cycling learning rates [34]. The default initial learning rate of ${10}^{-3}$ was applied. If not otherwise mentioned, ReLU was used as activation function ${\sigma }^{\left(1\right)}$, identity as activation function ${\sigma }^{\left(2\right)}$. Since the presented NODEs are tailored to PK scenarios and thus the functions that the NN has to learn are very limited compared to other applications, NNs with only one hidden layer were used and the number of hidden neurons was set to ${n}_{Hid}=20$. This setup exceeds the required number for most PK scenarios. Since a general NODE concept is presented that should not be project specific, no hyperparameter tuning concerning learning rate or NN structure was performed. Graphs were generated using the package Matplotlib [35]. In Appendix A5 a shortened but documented code example fitting an NODE to data from a one-compartment IV ODE model is presented and in the Supplemental Material the full code is deposited.

## Results

In the following sections, the previously elaborated NODE structures, concepts and setups for applications were utilized in various PK scenarios. First, an NODE was applied to fit multi-compartmental behavior. Second, an application of an NODE to fit data with absorption and delayed absorption is presented. Third, data based on an IV infusion administration was fitted. Fourth, the capability of NODEs to fit highly intricate PK profiles originating from non-linear behavior such as TMDD [36–38] is illustrated. Finally, the application of NODEs with known mechanistic parts combined with NNs are presented.

### Application of an NODE to data with a multi-compartmental behavior

Consider the typical PK situation of multi-compartmental behavior, e.g., data consisting of a distribution and elimination phase. Pooled training was performed on data simulated with the two-compartment IV ODE model at 5 time points for two training doses ($d$ = 1 and $d$ = 10). The same model parameters as in the previous paragraph were applied with the additional peripheral transfer rates ${k}_{12}$ = 0.2 and ${k}_{21}$ = 0.2.

**Fig. 6:**
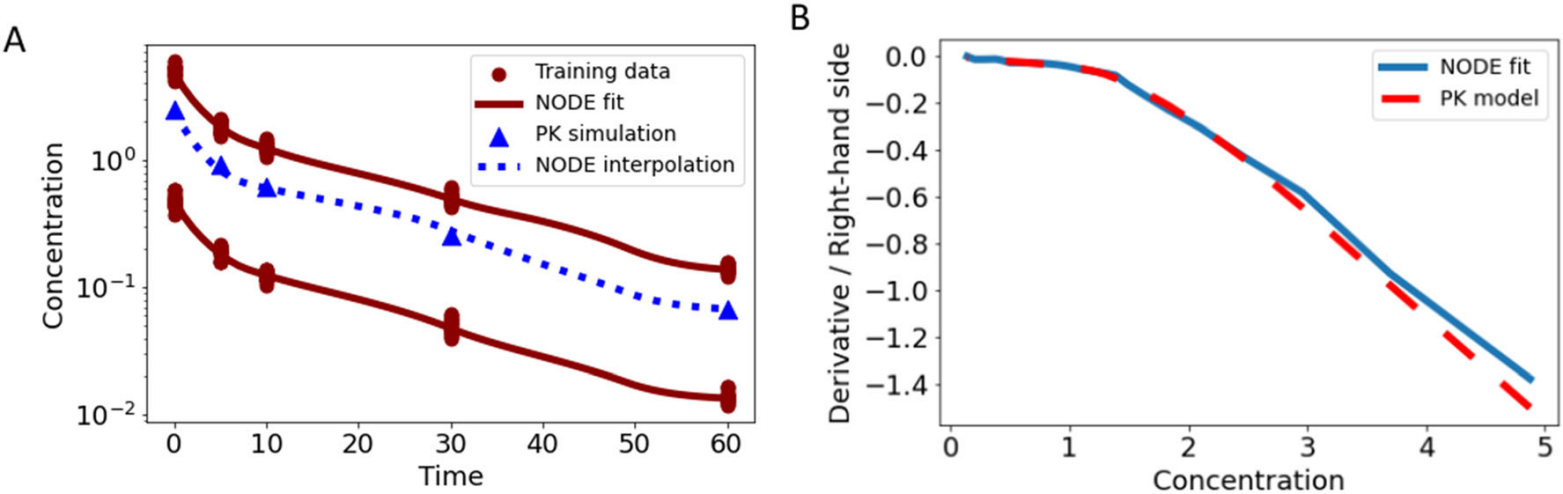
Data applied for training (red circles) with doses $d$ = 1 and $d$ = 10 and simulation (blue triangles) with $d$ = 5 were generated with a two-compartment IV ODE model, see panel **A**. Furthermore**,** fits of the trained NODE Eq. (6) for the two training doses (solid red line) and simulation (dashed blue line) are presented. In panel **B**, the corresponding derivative versus state plot indicates that the NODE learned the two-compartmental behavior from the training data

Data was well fitted with the NODE Eq. (6) and a simulation for an unseen dose $d$ = 5 coincide with the original ODE, see Fig. 6A. As demonstrated in Fig. 6B, the original mechanism was well learned.

### Application of an NODE to data with absorption and delayed absorption

Drugs often have an absorption phase, e.g., due to orally or subcutaneous administration. Occurrence of absorption can also be delayed. Pooled training was performed on data simulated with a one-compartment PO ODE model without (7 time points) and with transit compartments (10 time points) for two training doses ($d$ = 5 and $d$ = 15). Model parameters were as in the previous examples but now additionally with ${k}_{a}$ = 0.2 and in case of transit compartments we set ${n}_{tr}$ = 4 or 8 with ${k}_{tr}$ = 0.1.

**Fig. 7:**
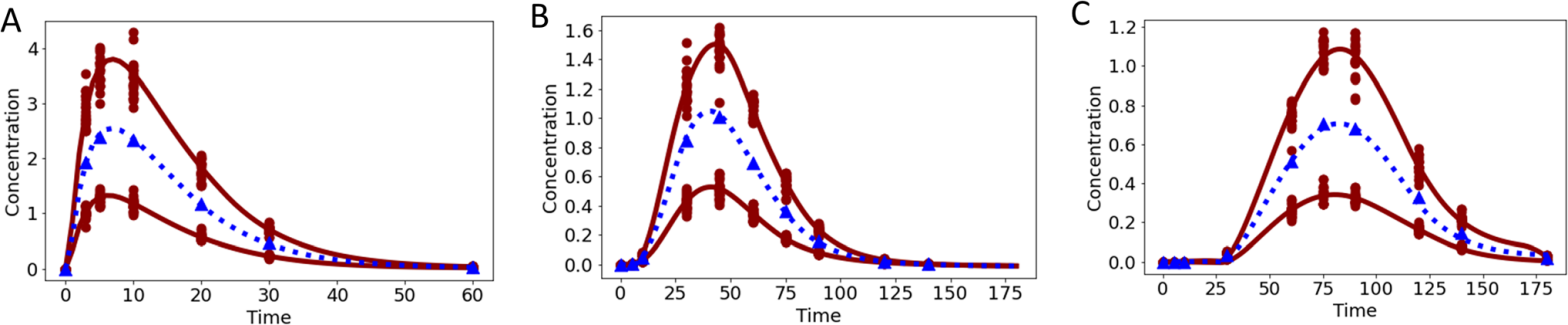
Data applied for training (red circles) with doses $d$ = 5 and $d$ = 15 and simulation (blue triangles) with $d$ = 10 were generated with a one-compartment PO ODE model either without transit compartments (panel **A**), 4 transit compartments (panel **B**) or 8 transit compartments (panel **C**). Fits (solid red line) and simulation (dashed blue line) of the NODE Eqs. (7, 8) are presented

Data was well fitted with the NODE Eqs. (7, 8), see Fig. 7A–C. In all three examples, NODE simulations for an unseen dose $d$ = 10 matched well with simulation from the original ODE.

### Application of an NODE to data with an IV infusion administration

We consider simulated data based on a one-compartment IV infusion ODE model. Pooled training was performed on data for two infusion rates (${k}_{in}$ = 1 and ${k}_{in}$ = 10) with an infusion time of ${t}_{inf}$ = 6 and ${k}_{el}$ = 0.1.

**Fig. 8:**
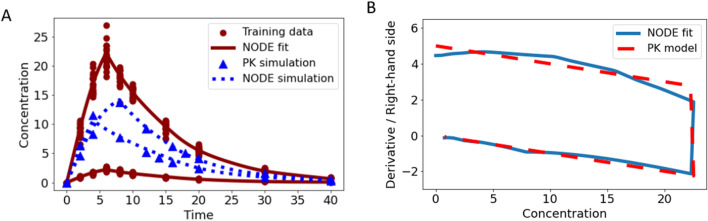
Data applied for training (red circles) and simulation (blue triangles) generated with a one-compartment IV infusion ODE model, see panel **A**. Furthermore, fits of the trained NODE Eq. (9) for the two training doses (solid red line) and simulations (dashed blue lines) are presented. In panel **B**, the corresponding derivative versus state plot indicates that the NODE learned the mechanism well

Data was well fitted with the NODE Eq. (9), compare Fig. 8A. The trained NODE could also simulate data for unseen infusion rates (${k}_{in}^{(1)}$ = 7 and ${k}_{in}^{\left(2\right)}$ = 5) with unseen infusion times (${t}_{inf}^{\left(1\right)}$ = 4 and ${t}_{inf}^{\left(2\right)}$ = 8) if the concentrations and total doses were in range of the training concentrations. The derivative versus state plot illustrates the properly described derivative of the model, including the drop in the derivative when infusion stops at ${t}_{inf}$ = 6, compare Fig. 8B.

### Application of an NODE to non-linear pharmacokinetics

Monoclonal antibodies often exhibit non-linear kinetics. The TMDD model in the original formulation [36–38] is an intricate ODE, consisting of three state variables. We consider an IV bolus administration and no distribution to a peripheral compartment. Pooled training was performed on data simulated with the original TMDD model at 7 time points for two training doses ($d$ = 50 and $d$ = 200). Model parameters of the original ODE were ${k}_{el}$ = 0.1, ${k}_{on}$ = 0.25, ${k}_{off}$ = 0.01, ${k}_{syn}$ = 0.5, ${k}_{deg}$ = 0.25 and ${k}_{int}$ = 0.1.

**Fig. 9:**
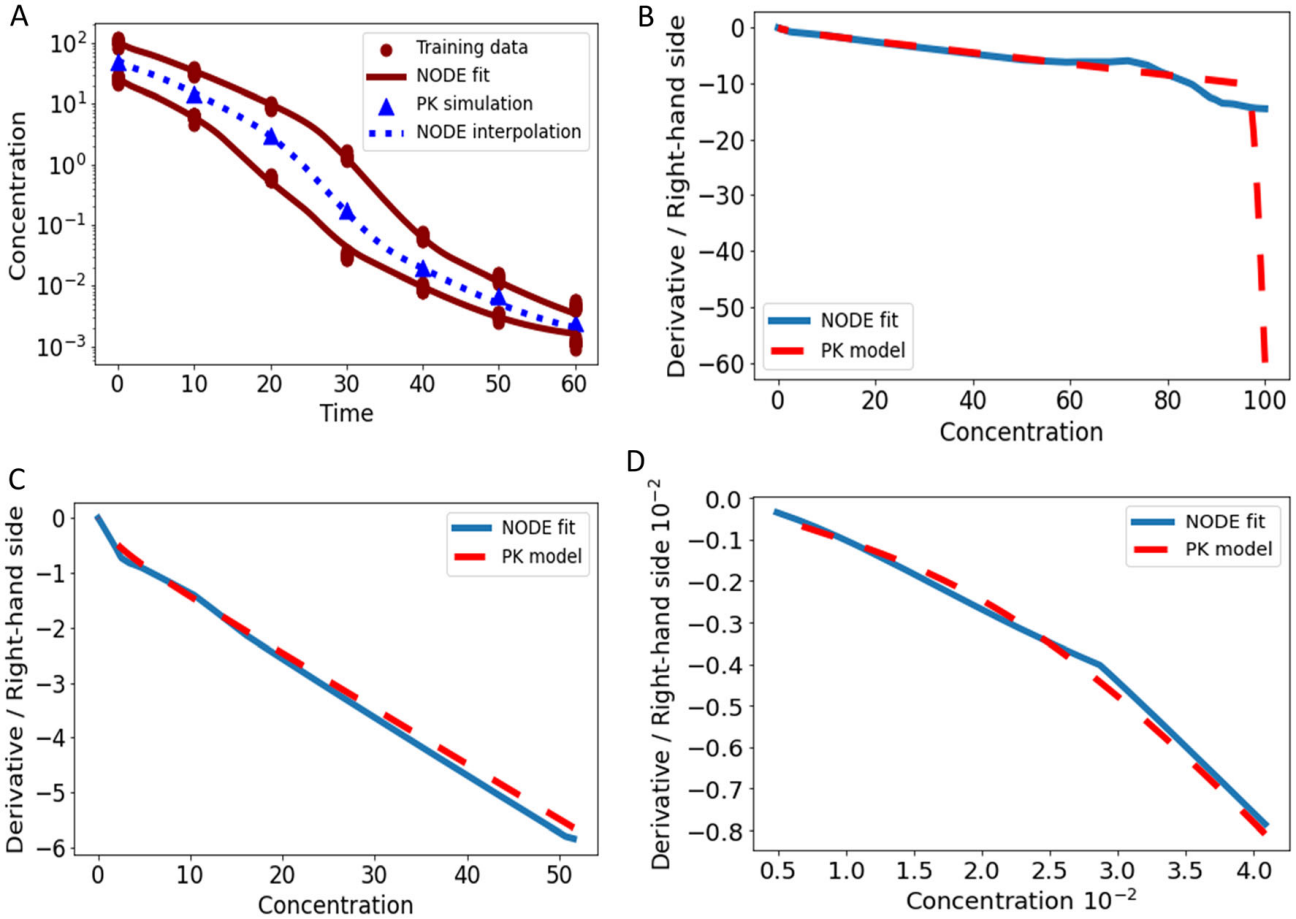
Data applied for training (red circles) with doses $d$ = 50 and $d$ = 200 and simulation (blue triangles) with $d$ = 100 were generated with the original TMDD model, see panel **A**. Furthermore, fits of the trained NODE Eq. (6) for the two training doses (solid red lines) and simulation (dashed blue line) are presented. In panel **B**, **C**, and **D**, the corresponding derivative versus state plot indicates that only mechanisms that are observable in the data can be learned. Since the measurement time-points are not dense enough to picture the initial rapid binding, this mechanism is not learned by the NODE, as can be seen in panel B. However, the linear elimination, transition, and terminal elimination phase were learned well

The s-shaped TMDD concentration–time curve, visible in semi-log scale, was well fitted with the NODE Eq. (6), shown in Fig. 9A. Note that the rapid binding of the ligand to the receptor in the original PK model was not captured by the NODE as the simulation time points are not dense enough to picture this drop, compare Fig. 9B. However, linear elimination phase, transition phase, and terminal elimination phase were well described by the NODE, compare Fig. 9C and D. NODE simulation for an unseen dose $d$ = 100 matched well with simulation from the original model. As observed concentrations range between 10^2^ and 10^−3^, the WMSE Eq. (15) was applied in this example.

### Application of an NODE with known mechanistic parts

**Fig. 10:**
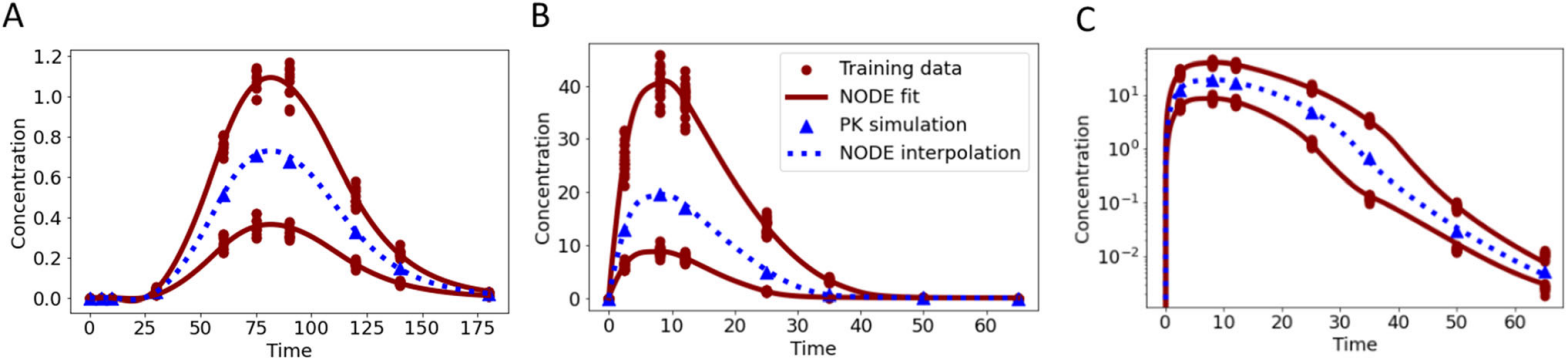
In panel **A**, fits (solid red lines) and simulation (dashed blue lines) of the NODE with Eqs. (10, 11) trained on data (red circles) generated with the one-compartment PO ODE model with 8 transit compartments for different dose levels is shown. In panels **B** and **C**, fits and simulation of the NODE with Eqs. (12, 13) trained on data generated with the original TMDD model with extravascular administration for different doses is shown in linear and in semi log scale, respectively

Here, we demonstrate the power of NODEs to leverage prior knowledge and assumptions from classical PK modeling. In a first example, we assume, that a drug is eliminated linearly with a first-order elimination rate. However, no assumptions about the absorption of this drug were made. Pooled training was performed on data from the one-compartment PO ODE model with 8 transit compartments for the NODE in the previous example, compare Fig. 7C. This data was fitted with the NODE Eqs. (10, 11), compare Fig. 10A. In a second example, first-order absorption together with a complex, non-linear elimination is assumed. For this scenario, pooled training was performed on data generated with the TMDD model with extravascular administration for two doses ($d$ = 50 and $d$ = 200). The same TMDD parameters were used as in the IV TMDD example with an absorption rate constant ${k}_{abs}$ = 0.15. The NODE Eqs. (12, 13) was fitted to the data and the trained NODE was applied to simulate data for an unseen dose $d$ = 100, compare Fig. 10B and C.

## Discussion

In this section, we discuss developed concepts and evaluated applications of NODEs in pharmacometrics with the special focus on PK analyses.

In the “Theoretical” section, we presented concepts for our low-dimensional NODEs. We introduced NODEs, where the explicit function, i.e., the right-hand side, of an ODE is substituted with an NN. In contrast to classical PK modeling, where an explicit mechanism is applied, NODEs are mainly data-driven and therefore differ substantially in two points. First, the classical approach allows to obtain information about PK properties, e.g., interpretable PK parameters are derived, such as clearance. An NODE does not provide similar information. Second, all mathematical properties of the PK model are known or can be derived from dynamical systems theory. Therefore, it is qualitatively known how the developed PK model will behave for simulations, e.g., for different doses or varying model parameters where no data is available. With NODEs, the application for simulations is more restricted, as discussed later.

To make NODEs applicable for various PK scenarios, we developed a tailored concept, including NODE structures derived from known principles of PK modeling. Since the mechanism is solely learned from the data, the necessary multi-dimensionality originating from modeling the underlying mechanism is not known. Therefore, a key concept in the presented NODE approach is the reduction of multi-dimensional systems to a non-autonomous one-dimensional system, compare Eq. (5). Motivated by Appendix A1–A4, the presented basic NODE structure was further adjusted and the inputs concentration and time were separated into two different NNs, compare Eq. (6). As a result, the general NODE has the advantage that the question about the number of necessary peripheral compartments does not exist anymore. Further, an absorption process was included into the NODE. Since, it is known whether absorption process takes place or not, we extended the NODE structure with an additional compartment for the absorption, compare Eqs. (7, 8). The time-dependent NN in Eqs. (7, 8) allows to also characterize strongly delayed absorption processes. With the resulting separation of absorption and distribution processes in the NODE, the required NN size might be reduced and thus the potential risk of overfitting can be mitigated. Again, as a result, the typical question about the number of transit compartments [39] does no longer exist with this NODE. This approach might impact cases when one state influences the dynamics of another state, e.g., when biliary excretion of a drug into the absorption compartment is observed. Since this work aims at giving a basic insight into NODEs, no such cases were considered. Summarizing, the proposed NODEs are partially motivated by PK principles from linear multi-dimensional compartmental ODE models. However, we remark that these principles are also applied in a non-linear NN setting. We also scratched on the surface of combining partially known mechanisms with an NN. This further reduces the complexity of the NODE and the number of parameters in the NODE drastically. Hence, the potential risk of overfitting and thus the amount of training data required might be reduced.

In the “Methods” section, we presented a setup for applications of our NODE structures in pharmacometrics. Hence, we investigated typical NN related aspects such as the phenomenon of overfitting and extrapolation to unseen data, which are unfortunately inherited when applying NNs, also in the context of NODEs. First, while an NODE is a much more flexible approach than a classical ODE, it also results in an increased risk of overfitting and the potential inaccuracy when performing simulations for unseen data. For both problems we suggested a practical solution that proves itself in all scenarios discussed in this manuscript. We demonstrated, that with pooled training of the NODE, overfitting can be reduced, and the NODE learns the underlying mechanism instead of individual PK curves. Since we present a general approach not related to a specific project, we did not perform hyperparameter tuning and thus no cross validation. In addition, we assumed PK datasets to be rather small in general and the separation into training and validation set might even negatively impact the model. Second, we illustrated, that NODEs can perform simulations for unseen data, if the simulations lay in the range of the training data, i.e., the NODEs are able to interpolate between two training doses. However, the inherent inability of NNs to make reasonable simulations for data outside of the observed range represents a clear limitation of NODEs compared to classical modeling approaches. Although we focus on PK analysis, the presented solutions in the “Methods” section are of a broader applicability for general pharmacometrics.

In the “Results” section, we utilized previously elaborated concepts and setups for application of our NODEs in various PK scenarios. First, our NODE structure could describe well PK data with IV bolus administration. Due to the high flexibility, our NODE structure can be applied to PK data with multi-compartmental behavior or intricated data such as PK data generated by a TMDD model. Second, the structural changes to apply an NODE with an absorption compartment accurately fitted PK data from an extra vascular administration including delayed absorption. Additionally, the NODE structure could also be adjusted to IV infusion administration. Finally, the application of an NODE structure with a combination of known mechanistic parts and an NN can simplify the fit of intricated data, such as data with TMDD behavior and extra vascular administration. Incorporating prior knowledge mechanistically could allow to reduce the flexibility of NODEs, e.g., compare Eqs. (12, 13) with Eqs. (7, 8). Therefore, this might also decrease the risk of overfitting. In all scenarios, the NODEs not only fitted well the PK data, but they were able to perform accurate simulations for unseen doses within the dose range observed in the training data.

One limitation of the presented case-studies is that we only considered single-dose scenarios. However, due to the implementation of our NODEs, a transition to multi-dose scenarios would be possible with some adjustments. However, this requires further investigation and will be covered in a separate paper. Further limitations of the presented work are that no covariates were included and that only average fitting was performed, i.e., no inter-individual variability was taken into account. Both limitations are topics for future work.

A general consideration with NODEs is that with very sparsely sampled data, no information is available between measurements. In this case, NODEs cannot learn the entire dynamics and might provide unrealistic predictions in between measurement time-points while still give good predictions for the measurements. Consequently, NODEs might not be applicable straightforward in such scenarios.

Overall, we conclude that NODEs substantially differ from classical PK modeling and may not substitute the task of a classical PK analysis, where e.g., gaining information about PK properties is one essential part. We also emphasize that we considered a specific representation of the NN, namely, only one hidden layer, a small number of neurons, a one-dimensional form of our NODE structure, and specific activation functions for basic applications. A relaxation of this representation might even increase the flexibility of NODEs resulting in successful modeling of much more complex phenomena, such as drug-drug interactions or metabolites with competing elimination. Hence, the presented applications are not exhaustive and further research work is warranted to expand use of NODEs to other pharmacometrics situations.

Based on the present evaluation of NODEs applications in this research work, we anticipated that future applications of NODEs will differ from typical PK analyses, e.g., they can be applied in situations where it is more important to perform automated modeling tasks and handle intricate profiles, and where no PK parameters, such as the clearance, should be obtained. We further speculate that the future of NODEs lies in the combination of mechanistic components with neural networks, particularly in the context of complex pharmacodynamic and physiology-based modeling and simulation.

In conclusion, this research work hopefully contributes a part to enhance understanding of how NODEs can be applied in PK analyses and illustrates the potential for NODEs in the field of pharmacology and pharmacometrics.

### Electronic supplementary material

Below is the link to the electronic supplementary material.

Supplementary file1 (PY 6 KB)

## Appendix

### A1: Reduction of a two-compartment model to a one-dimensional non-autonomous system

As motivation for the reduction of the multi-dimensionality to a one-dimensional non-autonomous system, we examine without loss of generality a two-compartment model

16 $$\frac{d}{dt}{x}_{C}=-{k}_{el}{\cdot x}_{C}-{k}_{12}{\cdot x}_{C}+{k}_{21}\cdot {x}_{P} , \,{x}_{C}\left(0\right)={x}_{C}^{0} ,$$

17 $$\frac{d}{dt}{x}_{P}={k}_{12}\cdot {x}_{C}-{k}_{21}\cdot {x}_{P} , \,{x}_{P}\left(0\right)=0 .$$

The solution of the peripheral compartment Eq. (17), see e.g., Gibaldi [40], can be substituted in Eq. (16) resulting in

18 $$\frac{d}{dt}{x}_{C}=-{k}_{el}\cdot {x}_{C}-{k}_{12}\cdot {x}_{C}+{k}_{21}\cdot {x}_{C}^{0}\cdot \left(\frac{{k}_{12}}{\beta -\alpha }\cdot \left(\mathrm{exp}\left(-\alpha \cdot t\right)-\mathrm{exp}\left(-\beta \cdot t\right)\right)\right), {x}_{C}\left(0\right)={x}_{C}^{0},$$

with the usual meaning for $\alpha$ and $\beta$, compare e.g., Gabrielsson [4]. Hence, we see the concentration and time-dependency in Eq. (18) which motivates Eq. (6).

### A2: Reduction of a system with multiple transit-compartments to a one-dimensional non-autonomous absorption system

Consider a transit compartment structure [39, 41] for an absorption delay, and we study without loss of generality a two transit compartment situation:

19 $$\frac{d}{dt}{x}_{1}=-{k}_{t}\cdot {x}_{1} , \,{x}_{1}\left(0\right)=\frac{d}{V} ,$$

20 $$\frac{d}{dt}{x}_{2}={k}_{t}\cdot \left({x}_{1}-{x}_{2}\right) , \,{x}_{2}\left(0\right)=0 ,$$

21 $$\frac{d}{dt}{x}_{A}={k}_{t}\cdot {x}_{2}-{k}_{a}\cdot {x}_{A} , \,{x}_{A}\left(0\right)=0 ,$$

22 $$\frac{d}{dt}{x}_{C}={k}_{a}\cdot {x}_{A}-{k}_{el}\cdot {x}_{C} , \,{x}_{C}\left(0\right)=0 .$$

Substitution of the solutions for Eqs. (19, 20) in Eq. (21) results in

23 $$\frac{d}{dt}{x}_{A}={k}_{t}\cdot {k}_{t}\cdot \frac{d}{V}\cdot t\cdot \mathrm{exp}\left(-{k}_{t}t\right) -{k}_{a}\cdot {x}_{A }, \,{x}_{A}\left(0\right)=0 ,$$

24 $$\frac{d}{dt}{x}_{C}={k}_{a}\cdot {x}_{A}-{k}_{el}{\cdot x}_{C }, \,{x}_{C}\left(0\right)=0 ,$$

which motivates the structure in Eqs. (7, 8).

### A3: Dimension reduction for general PK models

A general linear PK model can be expressed as

25 $$\frac{d}{dt}x(t)=A\cdot x\left(t\right),$$

with $x=\left({x}_{1}, \dots , {x}_{N}\right)$, $A\in {\mathbb{R}}^{N,N}$ and $x\left(0\right)=\left(\frac{d}{V},0,\dots ,0\right)$. For the first compartment ${x}_{1}$ the following initial value problem can be obtained

26 $$\frac{d}{dt}{x}_{1}(t)=\sum_{j=1}^{N}{a}_{1j}\cdot {x}_{j}(t)={a}_{11}\cdot {x}_{1}\left(t\right)+\sum_{j=2}^{N}{a}_{1j}\cdot {x}_{j}\left(t\right)$$

and ${x}_{1}\left(0\right)=\frac{d}{V}$. Now we approximate the two terms in Eq. (26) by two NNs, one in $t$ and one in $x$, both with one-dimensional input and output. Since ${x}_{j}(t)=\frac{d}{V}\cdot {\overline{x} }_{j}\left(t\right)$, $j=1,\dots ,N,$ holds with $\overline{x }$(t) denoting the solution of Eq. (25) with $\overline{x }\left(0\right)=(\mathrm{1,0},\dots ,0)$ due to the linearity of Eq. (25) [see also e.g., Eq. (18)], we obtain

27 $${x}_{1}\left(0\right)\cdot {f}_{NN}^{t}\left(t\right) \sim {x}_{1}\left(0\right)\cdot \sum_{j=2}^{N}{a}_{1j}\cdot {\overline{x} }_{j}\left(t\right)= \sum_{j=2}^{N}{a}_{1j}\cdot {x}_{j}\left(t\right) ,$$

$${f}_{NN}^{c}\left({x}_{1}\right) \sim {a}_{11}\cdot {x}_{1}\left(t\right).$$

This results in a general NODE structure

28 $$\frac{d}{dt}{x}_{1}(t)={f}_{NN}^{C}\left({x}_{1}\right)+{x}_{1}^{0}\cdot {f}_{NN}^{t}(t), \,{x}_{1}\left(0\right)={x}_{1}^{0}=\frac{d}{V} .$$

A similar dimension reduction process is possible for nonlinear PK models. Consider

$$\frac{d}{dt}x\left(t\right)=f\left(x\left(t\right)\right)$$

with $x=\left({x}_{1}, \dots , {x}_{N}\right)$, $f: {\mathbb{R}}^{N}\to {\mathbb{R}}^{N}$ and $x\left(0\right)=\left(\frac{d}{V},0,\dots ,0\right)$. We obtain for the first component

$$\frac{d}{dt}{x}_{1}\left(t\right)={f}_{1}\left({x}_{1}\left(t\right),{x}_{2}\left(t\right),\dots ,{x}_{N}\left(t\right)\right)$$

with ${x}_{1}\left(0\right)=\frac{d}{V}$. Let $g\left(t\right)=\left({x}_{2}\left(t\right),\dots ,{x}_{N}\left(t\right)\right)$, then we have

29 $$\frac{d}{dt}{x}_{1}\left(t\right)={f}_{1}\left({x}_{1}\left(t\right),g\left(t\right)\right)=h\left({x}_{1}\left(t\right),t\right) .$$

However, for general nonlinear PK models, there is no additive structure, in contrast to linear PK models, compare Eq. (26).

### A4: Separation of concentration/state and time dependent NNs

Consider Eq. (5) in its simplest form with ${n}_{In}$ = 2, ${n}_{Out}$ = 1, and ${n}_{Hid}$ = 1, and with ${\sigma }^{\left(2\right)}\left(z\right)=z$. Then we obtain

30 $${f}_{NN}\left({x}_{C},t\right)={w}_{11}^{(2)}\cdot {\sigma }^{\left(1\right)}\left({w}_{11}^{(1)}\cdot {x}_{C}+{w}_{12}^{(1)}\cdot t+{b}^{(1)}\right)+{b}^{(2)} .$$

We observe that both inputs, state and time, drive the activation function ${\sigma }^{\left(1\right)}$. In our opinion, this introduces disadvantageous restrictions e.g., in PK analysis, because of three reasons: first, due to the different scales of the input variables (time is continuously increasing while concentration decreases with time), second, due to the restriction of negative weights in the time-dependent NN, and third because Eq. (18) in Appendix A1 and Eq. (23) in Appendix A2 illustrated that also in classical modelling concentration- and time-dependent terms are separated when the system is reduced to a one-dimensional system.

For comparison, consider the right-hand side of Eq. (6) in its simplest form with $d=V=$ 1. Then we obtain

$${f}_{NN}^{C1}\left({x}_{C}\right)+{f}_{NN}^{C2}\left(t\right)={\overline{w} }_{11}^{2}\cdot {\overline{\sigma }}^{\left(1\right)}\left({\overline{w} }_{11}^{1}\cdot {x}_{C}+{\overline{b} }^{1}\right)+{\overline{b} }^{2}+ {\widehat{w}}_{11}^{2}\cdot {\widehat{\sigma }}^{\left(1\right)}\left({\widehat{w}}_{11}^{1}\cdot t+{\widehat{b}}^{1}\right)+{\widehat{b}}^{2} .$$
